# Comparison of N- and C-terminally endogenously GFP-tagged WEE-1.3 strains in *C. elegans*

**DOI:** 10.17912/micropub.biology.000353

**Published:** 2021-01-14

**Authors:** Lourds M. Fernando, Kyrionna Golliday, Ruby Boateng, Anna K. Allen

**Affiliations:** 1 Department of Biology, Howard University, Washington DC USA; 2 The Jackson Laboratory, Bar Harbor, ME USA

## Abstract

We have generated a WEE-1.3 strain in *C. elegans *where**we have endogenously tagged the C-terminus with GFP*.* In this publication we demonstrate that this new strain exhibits the same expression localization pattern as the WEE-1.3 antibody and N-terminally endogenously GFP-tagged WEE-1.3 strain that have been previously published. We also show for the first time that endogenously tagging WEE-1.3 at either termini does not affect the reproductive function of the worms.

**Figure 1. Comparison of N- and C-terminally endogenously GFP-tagged WEE-1.3 strains:**
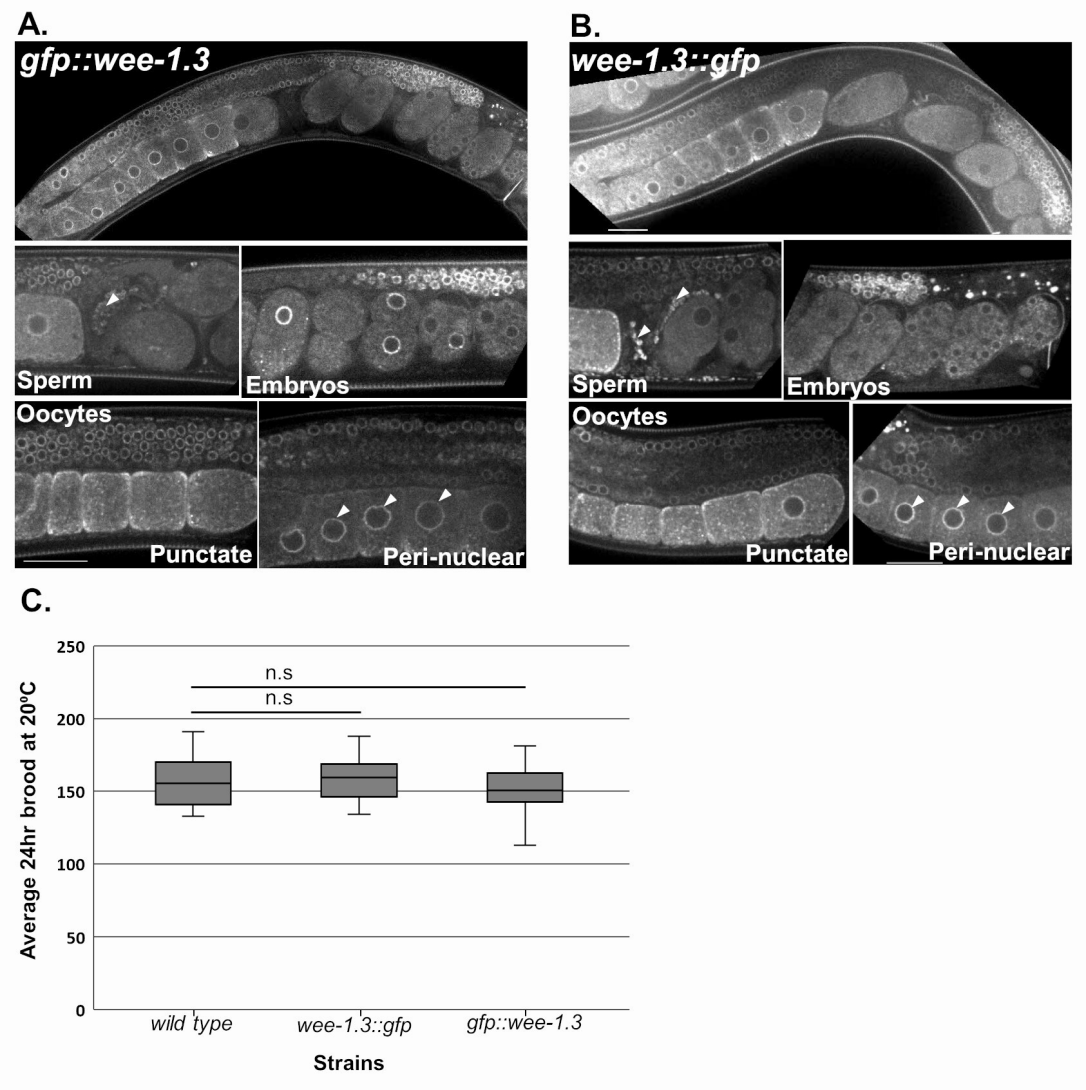
(A-B) Live confocal imaging of germ lines in w*ee-1.3(ana2[gfp::wee-1.3])* (A) and w*ee-1.3(ana8[wee-1.3::gfp])* (B) animals. Scale bars indicate 25µm. (C) 24-hour brood analysis comparison of endogenously tagged *gfp::wee-1.3* and *wee-1.3::gfp* strains compared to wild-type (N2) strain. n = 22-28 animals. Statistics were conducted by Student’s T-test, and n.s indicates not significant. Error bars represent the minimum and maximum values.

## Description

The Wee1/Myt1 family of kinases are important cell cycle regulators during mitosis and meiosis, and act by specifically phosphorylating certain amino acid residues and inhibiting their target genes. The *C. elegans* WEE-1.3 protein is a member of this family and has been shown to play an essential role in maintaining oocyte meiotic arrest in the nematode(Allen *et al.*, 2014; Burrows *et al.*, 2006)*.* A complete absence of WEE-1.3 results in embryonic lethality, while depletion of WEE-1.3 starting at the L4 larval stage results in precocious oocyte maturation and sterility (Allen *et al.*, 2014; Burrows *et al.*, 2006). Previously we have shown that WEE-1.3 is expressed throughout the soma and germ line of adult hermaphrodites and developing embryos via antibody staining, transgenic animals tagged with GFP at the N-terminus and C-terminus of WEE-1.3, and endogenously tagging the N-terminus of WEE-1.3 via CRISPR (Allen *et al.*, 2014; Fernando *et al.*, 2020). In these situations, the expression of WEE-1.3 is strongly perinuclear, but with cytoplasmic punctae that co-localizes with ER proteins in oocytes and embryos (Allen *et al.*, 2014; Fernando *et al.*, 2020). Here we have generated a homozygous *wee-1.3::gfp* strain (WDC8) where GFP is inserted at the 3′ end of the *wee-1.3* genomic locus via CRISPR/Cas9 endogenous genome editing. The endogenous C-terminal WEE-1.3::GFPstrain (WDC8) exhibits a perinuclear and cytoplasmic punctate localization expression pattern that is identical to that observed in the WEE-1.3 antibody stained germ lines, transgenic WEE-1.3::GFP animals and in endogenous *gfp::wee-1.3* (WDC2) strains (Figure 1A-B). This expression is ubiquitous throughout the soma, in the germ line from the distal tip to the proximal oocytes, in developing embryos, and in sperm stored in the spermatheca. Importantly, both N- and C-terminally endogenously GFP tagged WEE-1.3 strains have a normal 24-hour brood size compared to wild-type control animals implying that we have not disrupted the function of this important reproductive protein kinase by fusing the GFP protein directly to it (Figure 1C). Our data suggest that the new C-terminally endogenously GFP tagged WEE-1.3 strain represents wild-type WEE-1.3 expression and activity, and can be used to monitor endogenous WEE-1.3 levels and localization appropriately.

## Methods

The *wee-1.3(ana8[wee-1.3::gfp])* WDC8 strain was generated via CRISPR/Cas9 genome editing technology following the direct delivery method developed by the Seydoux laboratory (Paix *et al.*, 2017). Superfolder GFP sequence was inserted at the C-terminus immediately upstream of the stop codon (Pédelacq *et al.*, 2006). crRNA30 (5′- atttggatcatcaggcgacg – 3′) (Horizon Discovery Ltd) was used to guide the Cas9 to cut at the 3′ end of the *wee-1.3* gene. A PCR repair template was generated using pDONR221 containing Superfolder GFP and *wee-1.3* forward (oAKA431) and *wee-1.3* reverse (oAKA432) specific primers. oAKA431: 5′- gttttttttccagatgtcatttggatcatcaggcgacgAaGttt**ccaagggagaggagctctt**-3′

oAKA432: 5′ gcaaaaaataaatattcaacaatttttctgatttttgtgcattatta**cttgtagagctcgtccattc**-3′. The bold regions in the primers refer to the Superfolder GFP sequence. The uppercase letters in oAKA431 denote silent mutations that have been introduced to prevent recutting by crRNA30. Screening for successful edits was performed using the co-conversionmethod and *unc-58(e665)* as a marker (Arribere *et al.*, 2014). Successful edits were confirmed via PCR using forward primer oAKA88 (5′- gaataatgtgatcgacgaggctcc-3′) and reverse primer oAKA61 (5′- agtgcgatatggtcgagagg-3′). Multiple independent strains were generated, and each strain outcrossed five times before experiments were conducted.

Live imaging was conducted by placing 10-15 worms on a slide containing a 3% agar pad and 10uL of anesthetic (0.1% tricane and 0.01% tetramisole in 1x M9 buffer) then lowering a glass coverslip over the sample. Samples were imaged on a Nikon Ti-E-PFS inverted spinning-disk confocal microscope using a 60x 1.4NA Plan Apo Lambda objective, the 488 laser line, and an Andor iXon 897 EMCDD camera. All image editing was done using NIS-elements software.

For the brood assays, L4 hermaphrodites from each strain (N2, *gfp::wee-1.3*, *wee-1.3::gfp*) were placed onto *E. coli­*-seeded MYOB plates and then 16 hours after the L4 stage were singled out (1 worm/plate). The worms were grown at 20ºC, and after 24 hours, the adult mothers were discarded. For all plates, the progeny were counted 1 day after transferring the mother off of the plate. All progeny laid on the plate were counted when determining the brood; this includes eggs and developing larvae. Three independent trials were conducted for a total n ranging from 22-28.

## Reagents

N2

WDC2 *wee-1.3(ana2[gfp::wee-1.3])*

WDC8 *wee-1.3(ana8[wee-1.3::gfp])*

N2 is available from the CGC, WDC strains are available from the Allen Lab.

## References

[R1] Allen AK, Nesmith JE, Golden A (2014). An RNAi-based suppressor screen identifies interactors of the Myt1 ortholog of Caenorhabditis elegans.. G3 (Bethesda).

[R2] Arribere JA, Bell RT, Fu BX, Artiles KL, Hartman PS, Fire AZ (2014). Efficient marker-free recovery of custom genetic modifications with CRISPR/Cas9 in Caenorhabditis elegans.. Genetics.

[R3] Burrows AE, Sceurman BK, Kosinski ME, Richie CT, Sadler PL, Schumacher JM, Golden A (2006). The C. elegans Myt1 ortholog is required for the proper timing of oocyte maturation.. Development.

[R4] Fernando LM, Elliot J, Allen AK (2020). The Caenorhabditis elegans proteasome subunit RPN-12 is required for hermaphrodite germline sex determination and oocyte quality.. Dev Dyn.

[R5] Paix A, Folkmann A, Seydoux G (2017). Precision genome editing using CRISPR-Cas9 and linear repair templates in C. elegans.. Methods.

[R6] Pédelacq JD, Cabantous S, Tran T, Terwilliger TC, Waldo GS (2005). Engineering and characterization of a superfolder green fluorescent protein.. Nat Biotechnol.

